# Characterization of the Calcium Binding Protein Family in Zebrafish

**DOI:** 10.1371/journal.pone.0053299

**Published:** 2013-01-16

**Authors:** Vincenzo Di Donato, Thomas O. Auer, Karine Duroure, Filippo Del Bene

**Affiliations:** 1 Institut Curie, Centre de Recherche, Paris, France; 2 CNRS UMR 3215, Paris, France; 3 INSERM U934, Paris, France; 4 Centre for Organismal Studies Heidelberg, Im Neuenheimer Feld 230, University of Heidelberg, Heidelberg, Germany; University of Birmingham, United Kingdom

## Abstract

Calcium Binding Proteins (CaBPs), part of the vast family of EF-Hand-domain containing proteins, modulate intracellular calcium levels. They thereby contribute to a broad spectrum of biological processes – amongst others cell migration, gene expression and neural activity. In this study we identified twelve members of this protein family in zebrafish including one gene (*cabp4b*) currently not present in the zebrafish genome assembly. To gain insight into their biological functions, we carried out a detailed analysis of the expression patterns of these genes during zebrafish late embryonic and early larval development. We detected specific transcription for most of them in different neuronal cell populations including the neuroretina, the inner ear and the notochord. Our data supports potential roles for CaBPs during neuronal development and function and provides a starting point for genetic studies to examine CaBPs' function in these tissues and organs.

## Introduction

Calcium signaling is a fundamental process required for communication between neurons [1]. A local and rapid increase in calcium concentration within the pre- and postsynaptic specialization is required for transmission of information throughout the nervous system. The massive influx of calcium into the postsynaptic neuron triggers the activation of specific gene expression programs leading to long-term or short-term synaptic alterations [2], [3].

Since calcium-dependent gene transcription is involved in the control of crucial processes such as synapse development, maturation and refinement, it must be strictly regulated [4].

A broad spectrum of calcium sensing proteins acts as buffer for intracellular calcium, modulating and restricting the spatial and temporal impact of calcium as a second messenger. This gives these proteins a central role as regulators of the signaling pathways triggered or promoted by calcium.

Calcium binding typically elicits a conformational switch in the sensor. This switch leads to various interactions with downstream effectors and ultimately modulates neuronal activity [5]. For instance, the calcium binding protein Calmodulin (CaM) has been shown to bind the intracellular domain of the L-type voltage sensitive calcium channel and, upon calcium influx, to activate Ras/MAP Kinase signaling thus triggering gene transcription in the nucleus [6].

In mammals, the Calmodulin (CaM) superfamily, characterized by an EF-hand calcium binding motif, forms the largest class of calcium sensing proteins [7]. A related subfamily, the Calcium Binding Proteins (CaBPs) share a very similar domain organization with CaM and have been shown to have coevolved in vertebrate animals [8]. In humans the Calcium Binding Protein family is composed of six members: CaBP1, 2, 4, 5, 7 and 8. While the *cabp6* gene has not been identified, *cabp3* is most probably a pseudogene [9]. *Cabp7* and *cabp8* were alternatively referred to as *calneuron2* and *calneuron1* respectively in earlier studies [10], [11] and form a separate sub-family based on their sequence characteristics.

The expression pattern of single members of the CaBP family has been analyzed in human and mouse tissues (for a summary see Table 1.) but the localisation of the majority of them remains unknown. Furthermore, a few functional studies have been carried out, showing the involvement of CaBPs in the physiology of neural circuits. For example, *cabp4* and *5* were observed to be expressed in the retina where the respective proteins play a role in the modulation of visual stimuli, most likely by their interaction with voltage-gated Calcium channels [12] [13]. The specific expression of other Calcium binding proteins has been observed to be connected to a precise function [14] in their respective expression domains.

**Table 1:** Comparison of CaBP family member expression domains in mouse and zebrafish.

Gene in mouse	Expression domain	Reference	Gene in zebrafish	Expression domain
*cabp1*	Retina (INL* amacrine cells and subset of bipolars); Cerebellum	Haeseleer et al. (2000) [9]	*cabp1a*	Amacrine cells, heart, notochord
			*cabp1b*	Amacrine cells, otic vesicles, notochord
*cabp2*	Retina	Haeseleer et. al. (2000) [9]	*cabp2a*	Retina (bipolar cells), notochord
			*cabp2b*	Inner ear; neuromasts
*cabp4*	Photoreceptors	Haeseleer et. al. (2004) [12]	*cabp4a*	No specific expression
			*cabp4b*	Photoreceptors
*cabp5*	Retina (rod bipolar cells)	Haeseleer et. al. (2000) [9], Rieke et al. (2008) [13]	*cabp5a*	No specific expression
			*cabp5b*	Inner ear; Retina (bipolar cells); otic vesicles
*cabp7*	CA3** region of the hippocampus; entorhinal cortex; antero-dorsal and anteroventral thalamus; inferior and superior colliculus	Mikhaylova et. al.(2006) [11]	*cabp7a*	Dorsal thalamus; cerebellar plate
			*cabp7b*	Nucleus of medial longitudinal fascicle; reticular formation
*caln1* (*cabp8*)	Cerebellum; Hippocampus; Cortex	Wu et al. (2001) [10]	*caln1*	Pallium; Thalamus, pronephric duct; Retina (ganglion cell layer)
			*caln2*	Pallium; Thalamus; Retina (ganglion cell layer)

* inner nuclear layer.

** curnu ammonis.

In the following study we describe the expression patterns of all known Calcium binding proteins during the first five days of zebrafish development. We furthermore identified a new member of this gene family, *cabp4b* that is not part of the actual Zv.9 genome assembly.

## Results

### Phylogenetic analysis

**Figure 1:**
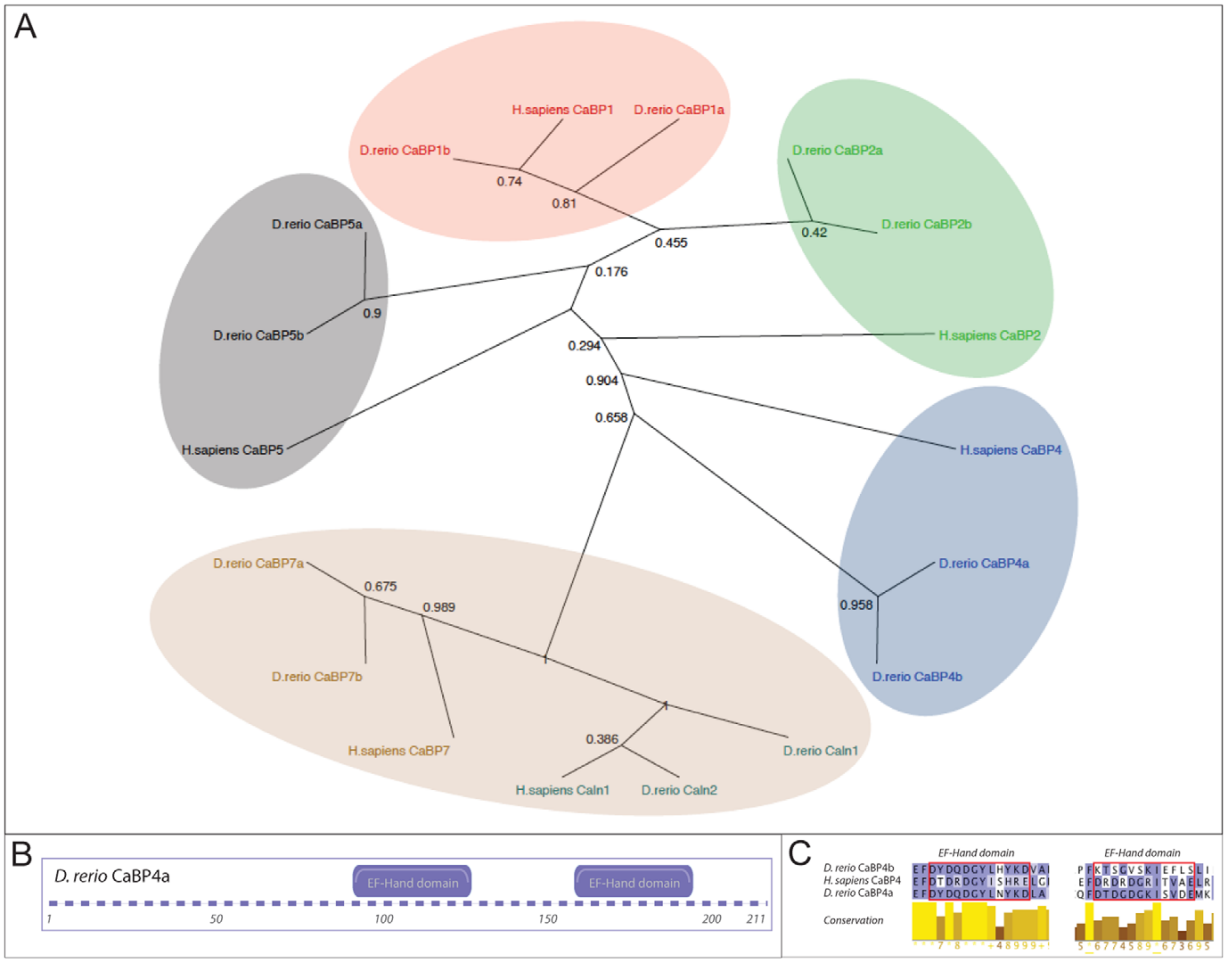
Phylogenetic analysis of the Calcium binding protein family in zebrafish. A) The phylogenetic tree of all human and zebrafish Calcium binding protein family members based on protein sequence. For all members there exist two paralogs in zebrafish per human paralog. *Cabp7* and *calneuron* (*cabp8*) form a separate subfamily (brown shading). B) Domain structure of CaBP4b as predicted by ExPasy (www.expasy.org). C) Protein alignment of the two EF domains of the human CaBP4 protein, the known zebrafish ortholog (*cabp4a*) and its newly discovered paralog (*cabp4b*). The blue color represents the Blosum62 score; high similarities are in dark blue while lower similarities are indicated in light blue. The first EF-Hand domain shows a higher degree of conservation than the second one.

To identify all Calcium binding protein family members in zebrafish we used the Ensembl orthology prediction tool. Based on their human orthologs we found two zebrafish paralogs for each gene (except *cabp4*). This is consistent with the commonly accepted theory that all teleosts underwent one additional round of whole genome duplication in evolution [15]. Surprisingly compared with the other 4 teleosts (medaka, takifugu, tetraodon, stickleback) zebrafish seemed to be the only species with just one paralog of *cabp4*. To identify a potential second zebrafish paralog for this gene we searched the NCBI expressed sequence tag collection for a suitable cDNA clone using the Tetraodon *cabp4* (2of2) cDNA sequence as bait. As the best hit, with 77% sequence identity, we identified a cDNA clone from a zebrafish retina cDNA library. This sequence was not present in the current Zv.9 assembly. When we aligned the cDNA clone with all other zebrafish *cabps* cDNA sequences it displayed the highest degree of conservation to *cabp4a* (data not shown). Using specific primers against the identified cDNA clone (IMAGE:4145466) sequence, we were able to amplify a 530 bp fragment from wild type Tupfel long fin cDNA. The online protein domain prediction tool Prosite from ExPasy assigned two EF-Hand domains to the unknown gene (Fig. 1C). Based on these results, we decided to call this cDNA clone *cabp4b*.

We subsequently calculated a phylogenetic tree based on the protein sequences of all *H. sapiens* and *D. rerio* Calcium binding proteins (for details see Material and Methods). As previously described [10] *cabp7s* and *cabp8s* (*calneurons*) form a separate subfamily (Fig. 1). *Cabp4* is closest related to this subtree followed by *cabp5*. Furthermore, both zebrafish *cabp5* and *cabp2* paralogs show a closer relationship to the zebrafish *cabp2* paralogs than to their human orthologs.

### In situ expression analysis

In order to determine the spatial distribution of calcium binding protein mRNA, we performed whole mount *in situ* hybridisation analyses with specific antisense probes.

### CaBP1s

**Figure 2:**
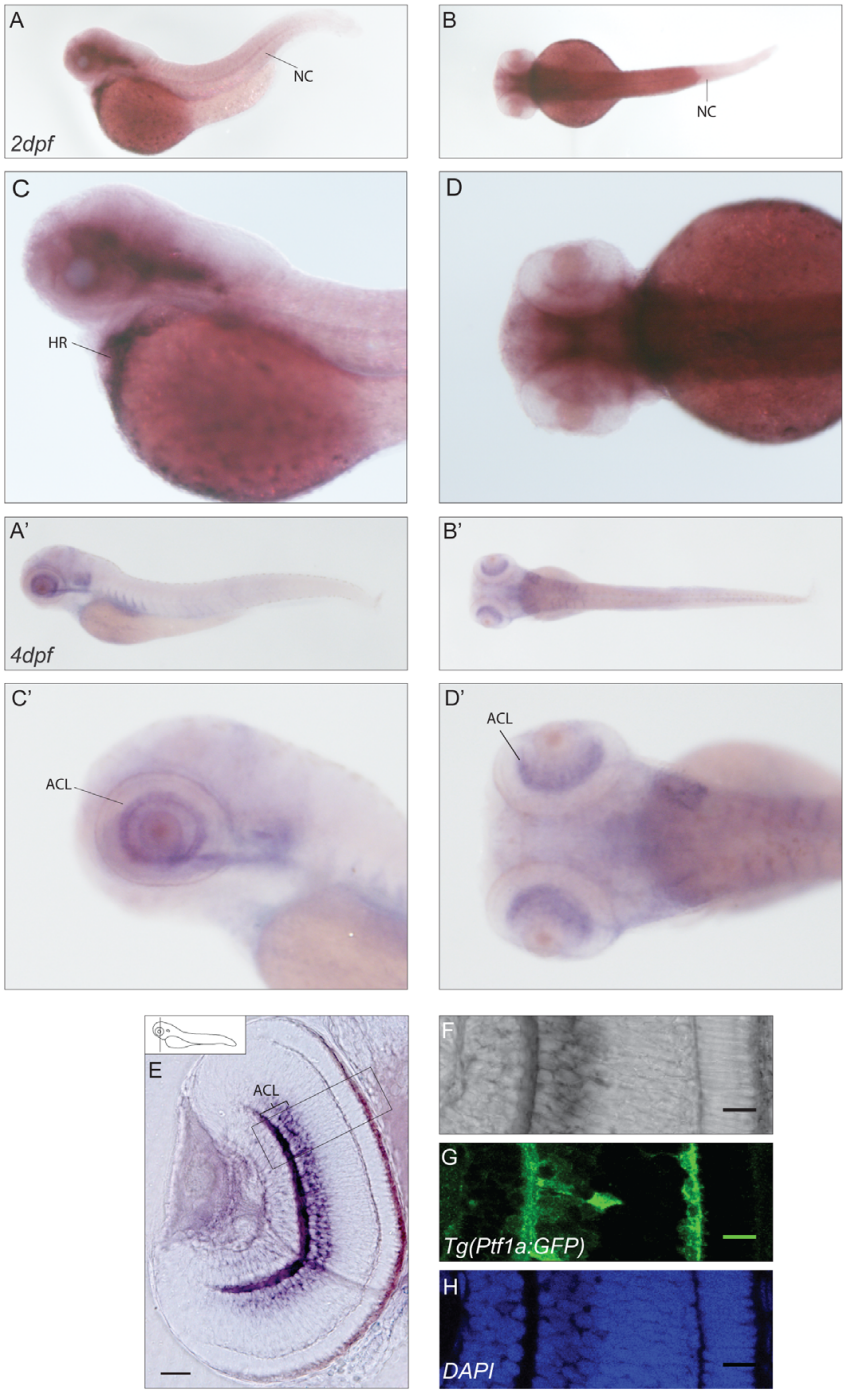
Expression pattern of *cabp1a*. (**A–D′**) mRNA expression of *cabp1a* in lateral (A–A′) and dorsal (B,B′) views of a 2dpf and 4dpf zebrafish embryo with higher magnifications (C–D,C′–D′). (**E–H**). Cross-sections of retinae in the Tg(Ptf1a:GFP) transgenic line show staining of *cabp1a* in the amacrine cell layer.(E) Epifluorescence picture of a sectioned retina. Scale bar: 50 µm (**F–H**) Confocal images of the area selected in E. (F) *In situ* signal in bright field, (G) GFP signal restricted to amacrine cells, (H) DAPI nuclear stain. Scale bar: 20 µm. HR: heart, NC: notochord, ACL: amacrine cell layer.

The expression pattern at different stages of development for both *cabp1a* and *cabp1b* is consistent with the data reported in the ZFIN website [16]. At 48hpf *cabp1a* exhibits a broad expression throughout the developing brain (Fig. 2, A–D) with additional expression in the notochord and the heart. 72hpf the staining of the antisense probe is restricted to the inner half of the inner nuclear layer (INL) of the retina (Fig. 2, A′–D′). To determine the precise neuronal type showing *cabp1a* expression we employed the zebrafish transgenic line Tg(Ptf1a:GFP) [17] (pancreas transcription factor 1 a) that has been shown to label the two inhibitory neuronal types (horizontal and amacrine cells) of the retina. Thereby, we were able to indicate amacrine cells as the most likely *cabp1a*-expressing population (compare Fig. 2 F–H). This observation is consistent with previous data by Wu et al. [10] where mRNA of the mouse *cabp1* ortholog was detected in amacrine cells of the mouse retina.

**Figure 3:**
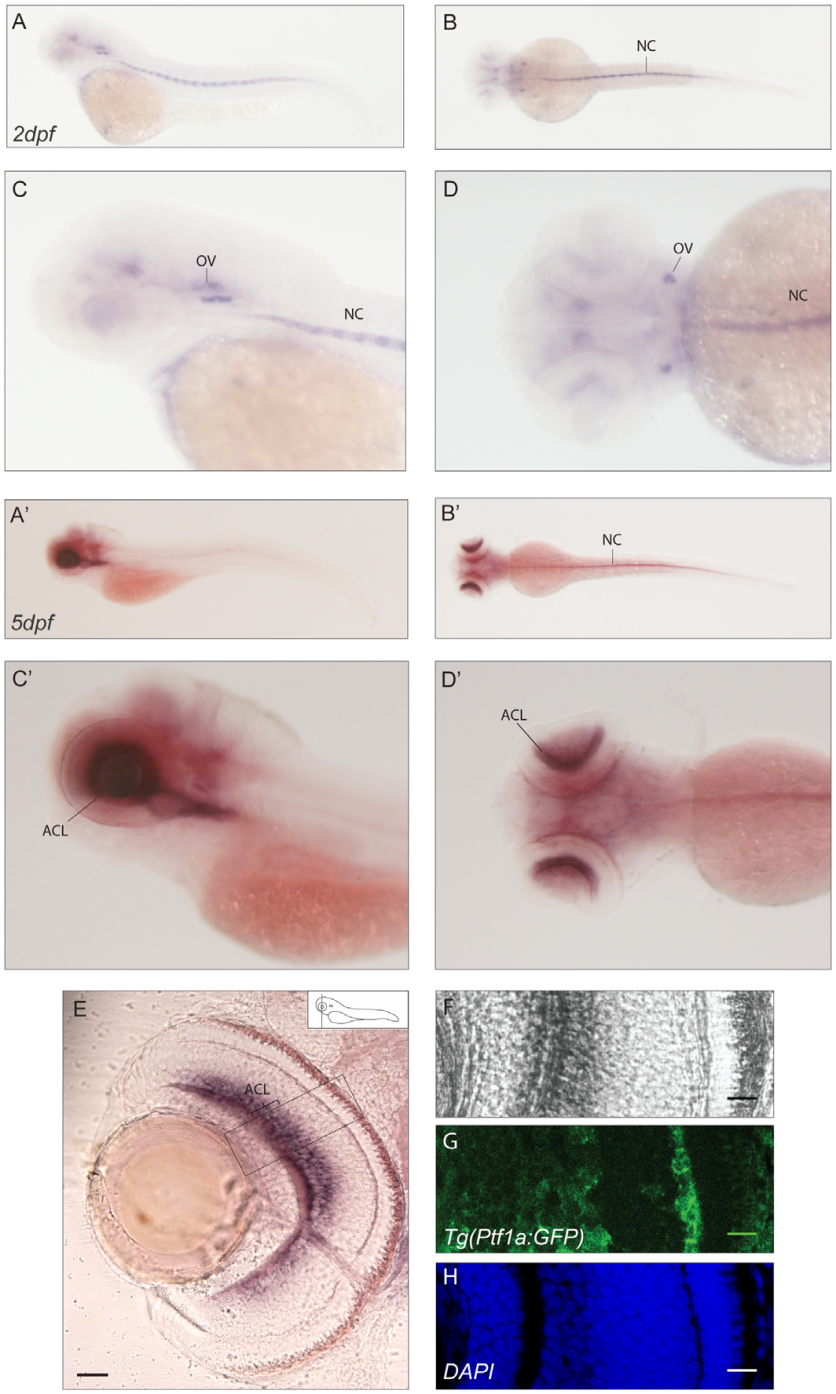
Expression pattern of *cabp1b*. (**A–D′**) *E*xpression in 2dpf (A–D) and 5dpf (A′–D′) zebrafish embryos: lateral (A,C–A′,C′) and dorsal (B,D–B′,D′) views. At 2dpf a signal in the otic vesicles and the notochord can be detected. At 5dpf expression in the notochord and the amacrine cell layer of the retina is seen. (**E–H**). Cross-sections of retinae in the Tg(Ptf1a:GFP) transgenic line show staining of *cabp1b* in the amacrine cell layer.(E) Epifluorescence picture of a sectioned retina. Scale bar: 50 µm (**F–H**) Confocal images of the area selected in E. (F) *In situ* signal in bright field, (G) GFP signal restricted to amacrine cells, (H) DAPI nuclear stain. Scale bar: 20 µm. OV: otic vesicle, NC: notochord, ACL: amacrine cell layer.

When the whole mount staining pattern of *cabp1b* was investigated, we could detect at 48hpf, a strong staining in the otic vesicles and along the notochord (Fig. 3, A–D). At 5dpf the strong expression of *cabp1b* in the notochord was maintained with an additional expression domain in the amacrine cells layer of the retina (Fig. 3, A′–D′). Comparison of *cabp1b* antisense probe signal with GFP in the Tg(Ptf1a:GFP) line strongly suggests expression in this cell type (Fig. 3, E–H).

### CaBP2s

**Figure 4:**
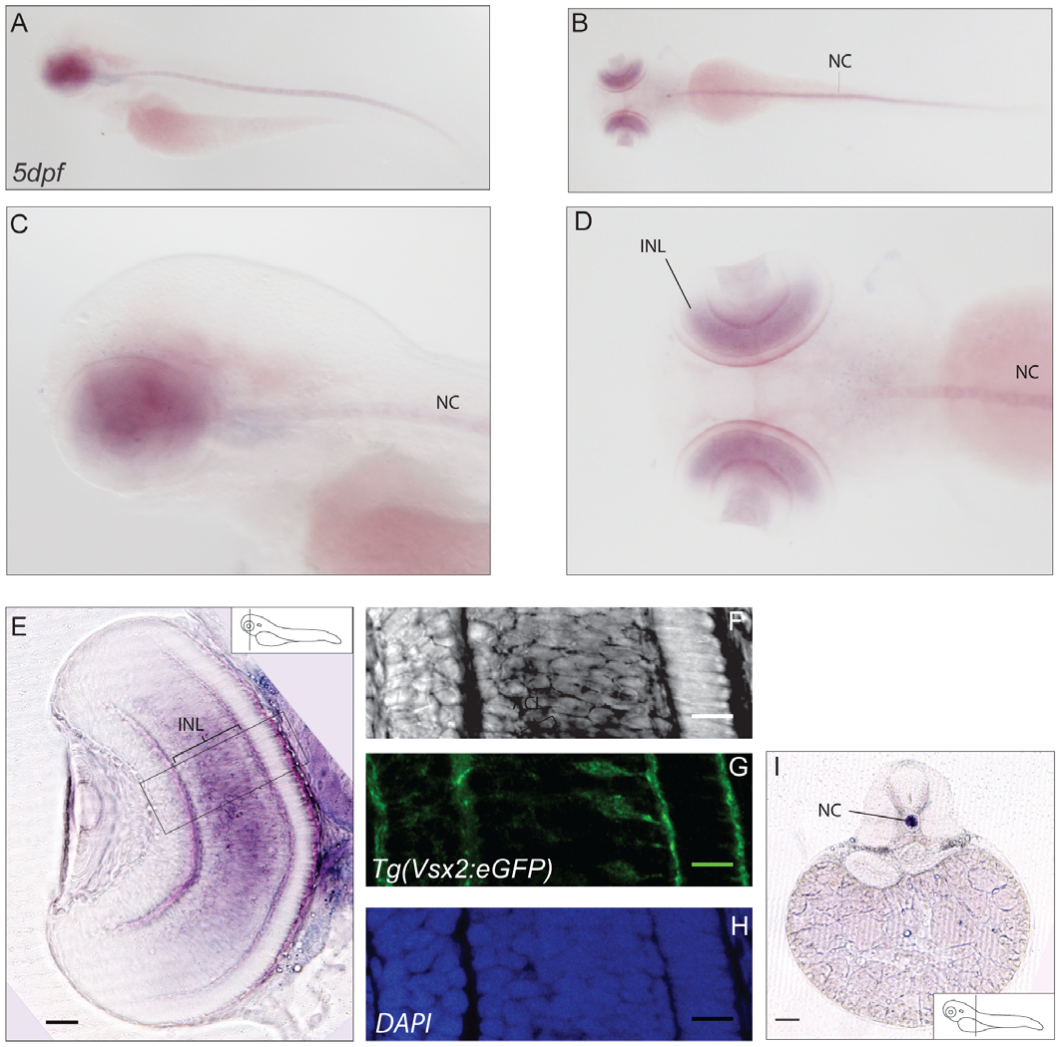
Expression pattern of *cabp2a*. (**A–D**) *In situ* hybridisation showing expression of *cabp2a* in 5dpf larvae. Lateral views (A,C) , dorsal views (B, D). *Cabp2a* antisense probe exhibits diffuse retinal localisation; a strong staining is also present along the notochord. (**E–H**) Cross-section of a retina in the Tg(Vsx2:GFP) transgenic line. (E) Epifluorescence image of a sectioned retina, showing prominent expression of *cabp2a* in the bipolar cell layer. Scale bar: 50 µm. (F–H) Confocal images of the area selected in E. (F) *In situ* signal in bright field, (G) GFP signal localized in bipolar cells, (H) DAPI. Scale bar: 20 µm (I) Cross-section exhibiting *in situ* staining in the notochord. Scale bar: 100 µm. INL: inner nuclear layer, NC: notochord.

Whole mount RNA expression analyses of *cabp2a* revealed no specific signal at early developmental stages. At 72hpf we could detect prominent expression in the retina and in the notochord (Fig. 4, A–D). To further clarify the identity of *cabp2a* expressing cells in the retinal tissue, we used the zebrafish transgenic line Tg(Vsx2:GFP) [18]. Vsx2, a homeodomain transcription factor, is initially expressed throughout the retinal epithelium, but is later enriched in a minor population of bipolar cells in the outer part of the INL. Localisation of GFP and the *in situ* signal (Fig. 4, E–H) show that *cabp2a* is expressed in the bipolar cells of the retina consistent with specific mRNA distribution of the mouse ortholog *cabp2* in the mouse retina and minor retinal component in other species [9].

**Figure 5:**
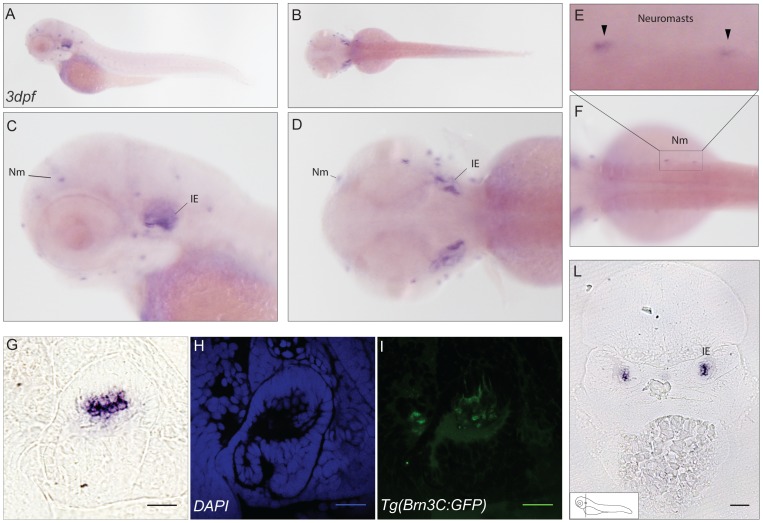
Expression pattern of *cabp2b*. (**A–D**) *In situ* hybridisation signal of *cabp2b* in 3dpf zebrafish embryos. Lateral (A) and dorsal (B) views of the embryo with higher magnification (C–D). Staining is restricted to hair cells in the inner ear and to the neuromasts. (F) View of stained neuromasts in the lateral line and (E) higher magnification of the selected area; arrowheads in E show the neuromasts. (**G–L**) Transverse section of the ear in the Tg(Brn3C:memGFP) transgenic line. (L) Epifluorescence image, showing strong expression of *cabp2b* in a subset of cells in the inner ear. Scale bar: 100 µm. (G) Higher magnification picture. (H–I) Confocal images. (H) DAPI signal, (I) GFP staining in the same domain as *cabp2b*. Scale bar: 20 µm. Nm: neuromast, IE: inner ear.

For *cabp2b*, both, the inner ear and neuromasts in the lateral line exhibit strong staining from 72hpf onwards (Fig. 5). The localisation in hair cells of both sensory organs was further supported by analyzing the expression in the Tg(Brn3C:memGFP) transgenic line [19]. In this line the GFP signal can be observed in a subset of retinal ganglion cells, hair cells of the inner ear and the lateral line neuromasts.

### CaBP4s

**Figure 6:**
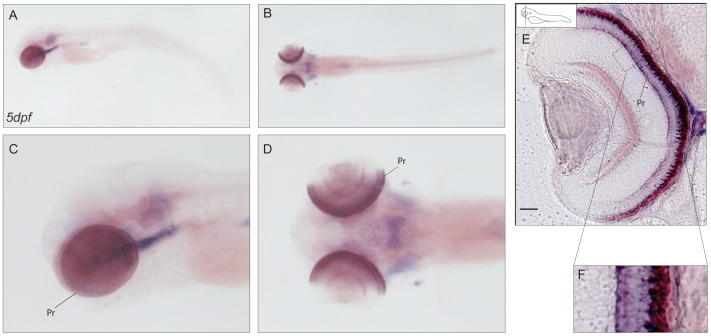
Expression pattern of *cabp4b*. (**A–D**) *In situ* localisation of *cabp4b* transcript in 5dpf larvae. Lateral (A–C) and dorsal views (B–D). A strong staining in the photoreceptor layer is detected. (**E–F**) Cross section of a retina (E) with zoom in to the photoreceptor layer (F). Scale bar: 20 µm. Pr: photoreceptors.

During the first five days of development no mRNA of the *cabp4a* gene could be detected. Similarly, the newly discovered *cabp4b* was not detected in any tissue within the first 72 hours post fertilization. Importantly, from day 4 onwards, a strong staining in the photoreceptor layer became visible (Fig. 6). This specific pattern of expression has also been described in mammals where *cabp4* was shown to play a crucial role in photoreceptor physiology [12]. The impairment of *cabp4* function triggers severe defects of vision like autosomal recessive night blindness [20].

### CaBP5s

**Figure 7:**
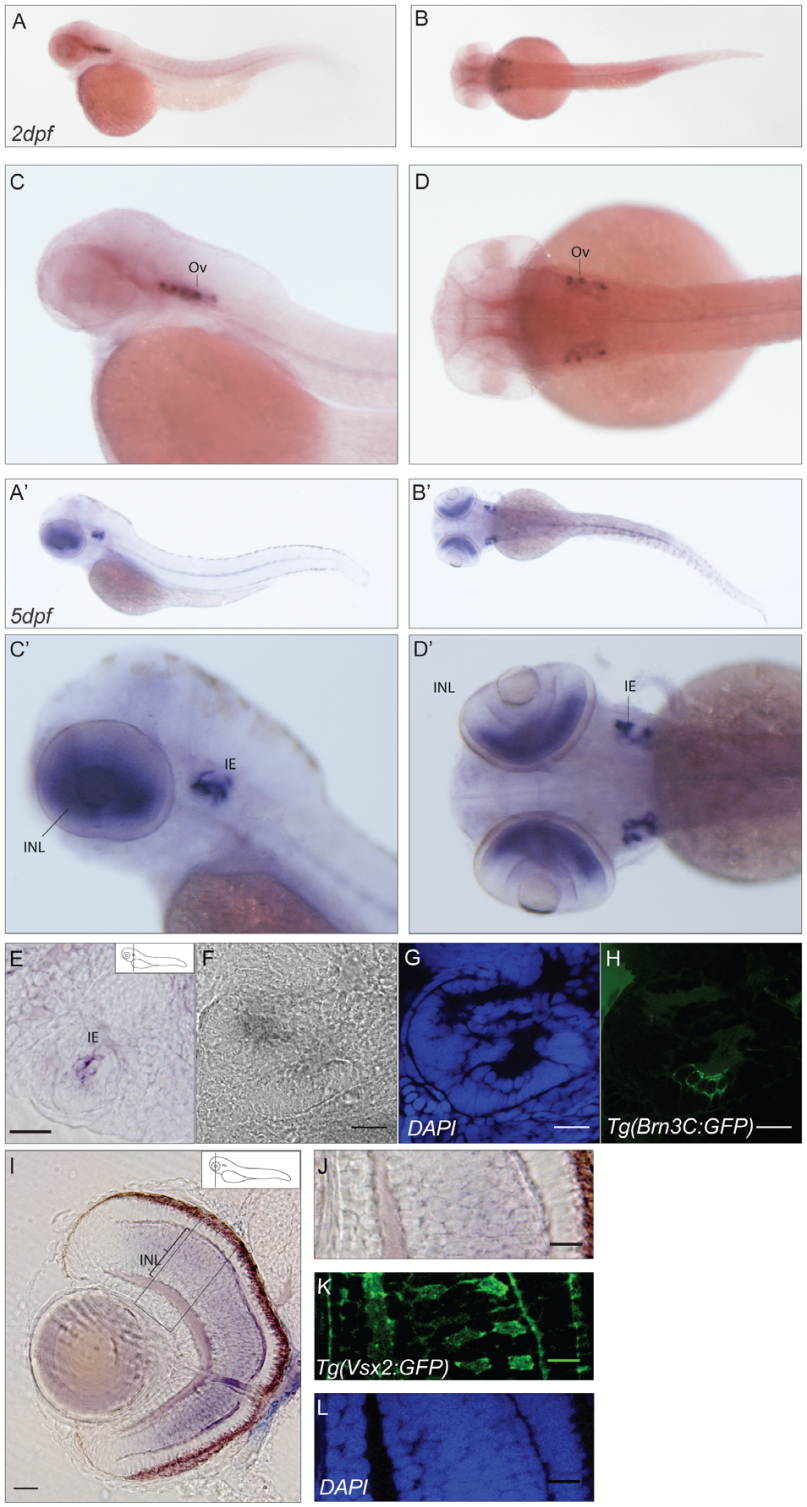
Expression pattern of *cabp5b*. (**A–D′**) *Cabp5b* expression in 2dpf (A–D) and 5dpf (A′–D′) zebrafish embryos: lateral (A,C–A′,C′) and dorsal (B,D–B′,D′) views. An intense staining is present in the inner ear and in the notochord at both stages of development. In 5dpf embryos, in addition, a strong signal is detectable in the retinal inner nuclear layer. (**E–H**) Sections of the ear in Tg(Brn3C:memGFP) transgenic line embryos. Epifluorescence image of a sectioned ear (E) and confocal images (F–H) showing strong expression of *cabp5b* in a subset of cells in the inner ear. Picture in bright field (F), DAPI (G), GFP (H). Scale bar: 20 µm. (**I–L**) Cross-sections of a retina in the Tg(Vsx2:eGFP) transgenic line. Epifluorescence image of a sectioned retina (I) and magnification (J), showing *cabp5b* antisense probe signal in the bipolar cell layer. Scale bar: 50 µm. (**K–L**) Confocal images. GFP signal localized in bipolar cells (K), DAPI (L). Scale bar: 20 µm. Ov: otic vesicle, IE: inner ear, INL: inner nuclear layer.

Endogenous *cabp5a* mRNA shows no expression in all the stages of development analyzed, while for adult eye tissue there was EST evidence reported [21]. For *cabp5b*, from 48hpf onwards, the expression pattern is consistent with the profile shown by Thisse *et al*., 2004^16^: the otic vesicles display a very prominent labeling, which is maintained in later stages (Fig. 7). In addition, in 5dpf larvae a strong signal is detectable in the retinal inner nuclear layer and in the notochord (Fig. 7, A′–D′). To identify the *cabp5b*-expressing cells, we assayed whole-mount *in situ* hybridisation in the zebrafish transgenic lines Tg(Vsx2:GFP) and Tg(Brn3C:memGFP). In the Vsx2 transgenic line we see expression of GFP and *cabp5b* in the inner nuclear layer of the retina, in what are most probably bipolar cells. On the contrary, *brn3C* and *cabp5b* expressing cells in the ear represent distinct populations. Interestingly, for mouse *cabp5*, a role in rod to bipolar cell signaling was proposed by Rieke et al. [13]. Upon loss of *cabp5* function, the rod-mediated responses of retinal ganglion cells were reduced which might reflect a change in the presynaptic function of rod bipolar cells.

### CaBP7s

**Figure 8:**
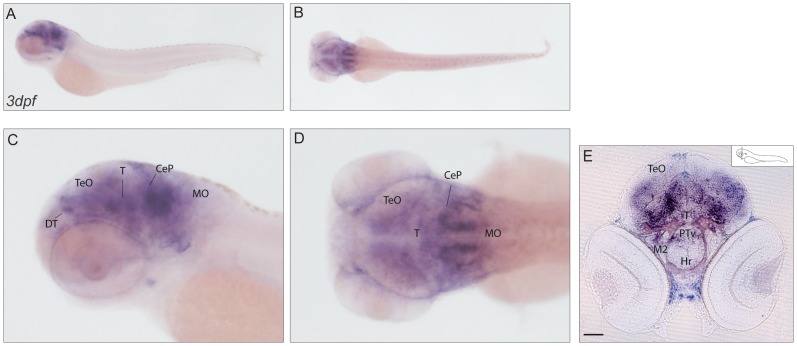
Expression pattern of *cabp7a*. (**A–D**) mRNA expression of *cabp7a* in lateral (A) and dorsal (B) views of 3dpf larvae with higher magnification (C–D). The signal of *cabp7a* antisense probe is widespread in the brain, with a stronger staining detected in the regions of the dorsal thalamus and the cerebellar plate. (**E**) Transverse section at the level of the eye, exhibiting diffuse expression of *cabp7a* in the developing brain. Scale bar: 100 µm. CeP: cerebellar plate; DT: dorsal thalamus; Hr: rostral hypothalamus; M2: migrated posterior tubercular area; MO: medulla oblongata; PTv: ventral part of posterior tuberculum; T: midbrain tegmentum; TeO: tectum opticum.

**Figure 9:**
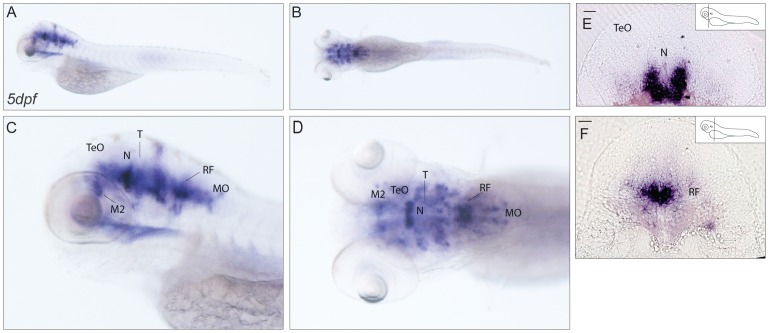
Expression pattern of *cabp7b*. (**A–D**) *In situ* localisation of *cabp7b* transcript in 5dpf larvae. Lateral (A,C) and dorsal views (B, D). A wide distribution of *cabp7b* mRNA in the brain was observed. (**E,F**) Transverse sections. Strongly stained cells are present in the midbrain, probably in the region of the nucleus of medial longitudinal fascicle (E) and in the hindbrain area anterior to the medulla oblongata most likely at the level of the reticular formation(F). Scale bar: 50 µm. M2: migrated posterior tubercular area; MO: medulla oblongata; N: nucleus of medial longitudinal fascicle; RF: reticular formation; T: midbrain tegmentum; TeO: tectum opticum.

Homolog to human *calcium binding protein 7* (ENSG00000100314, also known as *Calneuron2*), the two zebrafish genes *cab*p7a (annotated as *cabp7* 2of2, ENSDARG00000078272) and *cabp7b* (annotated as cabp7b, ENSDARG00000060846) were analyzed for their spatio-temporal expression pattern. *cabp7a* showed widespread expression throughout the brain from day 3 onwards with a prominent labeling detected in the dorsal thalamus region, as well as at the level of the cerebellar plate (Fig. 8). For *cabp7b*, the observed expression was more restricted to the midbrain and the hindbrain area anterior to the medulla oblongata (Fig. 9). Some regions, such as the migrated posterior tubercular area, the nucleus of medial longitudinal fascicle and the reticular formation, revealed a prominent staining. No expression in the dorsal parts of the brain such as optic tectum and cerebellum could be detected (Fig. 9, E, F).

### CaBP8s

**Figure 10:**
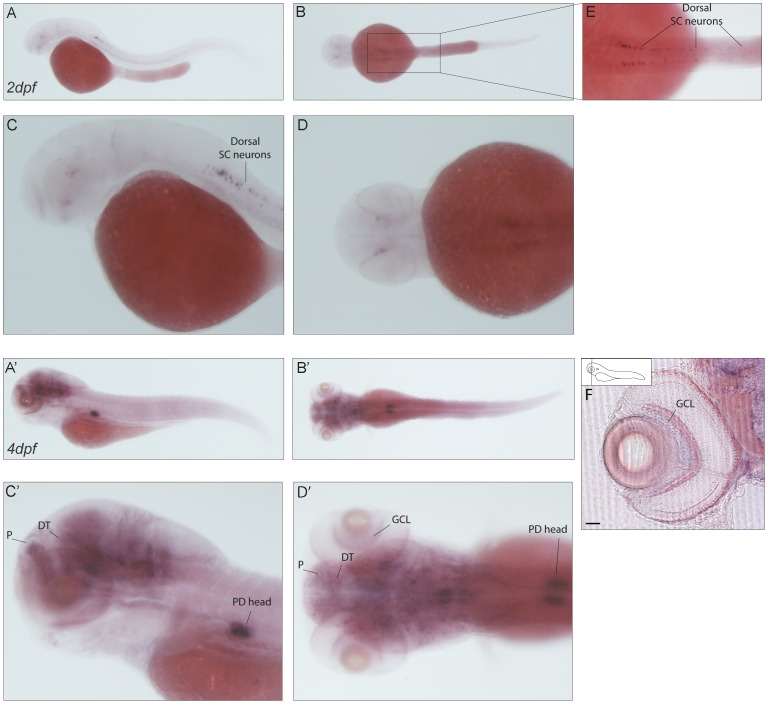
Expression pattern of *caln1*. (**A–D′**) *In situ* signal of *caln1* transcript in 2dpf (A–D) and 4dpf (A′–D′) larvae. Lateral (A,C–A′,C′) and dorsal views (B,D–B′D′). (E) Higher magnification shows specific staining in the dorsal spinal cord probably in Rohon Beard cells. (A′–D′) 4dpf whole mount embryos show diffuse signal in the brain and a specific expression in what is most probably the head of the pronephric ducts. (F) A transverse section through the retina shows expression in the retinal ganglion cell layer. Scale bar: 50 µm. SC neurons: spinal cord neurons, P: pallium; DT: dorsal thalamus; PD: pronephric duct; GCL: ganglion cell layer.

**Figure 11:**
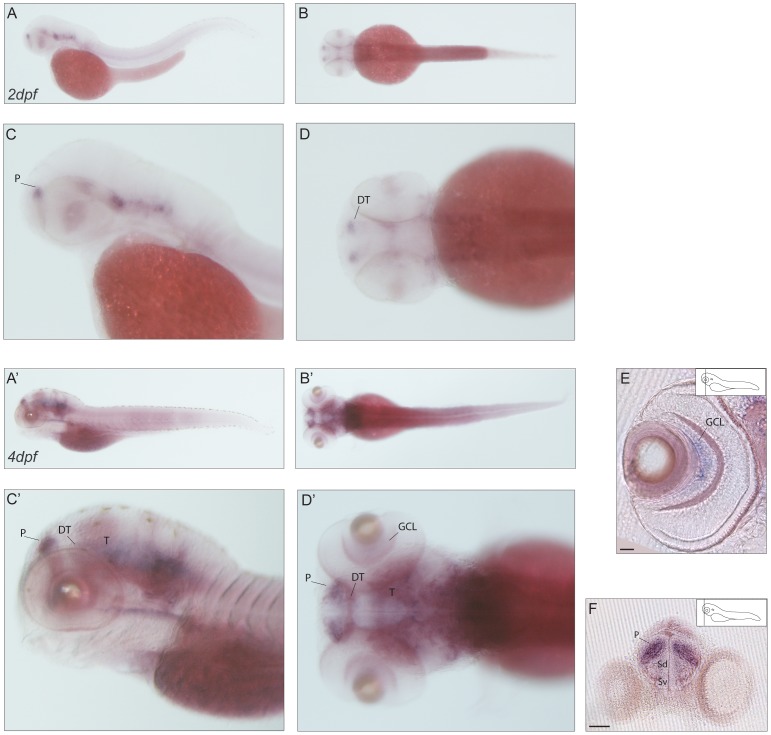
Expression pattern of *caln2.* (**A–D′**) mRNA expression of *caln2* in lateral (A,C–A′,C′) and dorsal (B,D–B′D′) views of 2dpf (A–D) and 4dpf (A′–D′) larvae with zoom in (C,D–C′,D′). The onset of *caln1* expression takes place within the first two days of embryonic development. (A–D) At 2dpf the *caln1* transcript localizes to the forebrain at the level of the subpallial region and in the caudal midbrain. (A-‘D’) The same expression profile seems to be maintained until day 4 of development. (E) Transverse section of the retina showing signal of *caln1* antisense probe in a subset of retinal ganglion cells in the central part of the ganglion cell layer. Strongly stained cells are present in the region of the telencephalic pallial domain (F). Scale bar: 50 µm. P: pallium; DT: dorsal thalamus; T: midbrain tegmentum; GCL: ganglion cell layer; Sd: dorsal division of subpallium; Sv: ventral division of subpallium.

*Calcium binding protein 8*, also known as *calneuron1*, has the two homologs *caln1* and *caln2* in zebrafish. Both of them are characterized by an early onset (between 24hpf and 48hpf) of expression (Fig. 10, A–D, Fig. 11, A–D). In 48hpf embryos, *caln1* exhibits a specific *in situ* hybridisation pattern, restricted to a population of primary sensory neurons - most probably Rohon Beard cells (Fig. 10, A–D). Analysis at later stages, 4dpf, reveals expression of *caln1* predominantly extending from the telencephalic pallial domain to the ventral thalamus region (Fig. 10, A′–D′). In addition, *caln2* is detected in 4dpf embryos in what is most probably the head of the pronephric ducts. The expression pattern observed for *caln2* seems to overlap with that of *caln1* at later stages - a very localized staining is detectable at 48hpf in the subpallial region and in the caudal midbrain (Fig. 11, A–D). *Caln1* and *2* are also detected in a subset of retinal ganglion cells – in the case of *caln2* localized at the center of the ganglion cell layer (Fig. 11, E).

## Discussion

Our work indicates that Calcium Binding Protein family members show very specific expression patterns in the course of zebrafish embryonic and larval development. Comparing our data with the published literature in mouse we observe a consistent conservation of most expression domains between the mammalian and the teleost clade (Table 1). In addition, we detected expression of *cabp2b* and *cabp5b* in hair cells of the inner ear, that has not been previously described in mice, and the neuromasts of the lateral line.

As a consequence of the extra genome duplication in teleosts, the two zebrafish orthologs either showed distinct expression patterns (i. g. *cabp2a* and *cabp2b*) or expression could not be detected for one of the two paralogs (i. g. *cabp1a* and *cabp1b*). The separate expression domains of the two paralogs suggest a possible subfunctionalization during teleost evolution [22]. This remarkable feature of the zebrafish system and its amenability to genetic manipulation will allow a detailed dissection of CaBP function in the different neuronal subpopulations where they are expressed.

In addition, we identified a new member of the *cabp* family, *cabp4b*, that is not part of the current zebrafish genome assembly. In humans, this gene has been related to different retinal pathologies such as the congenital cone–rod synaptic disorder and autosomal recessive night blindness.

Therefore, our findings open up the possibility to further dissect the molecular mechanisms underlying the pathology in humans using zebrafish as a model organism.

## Materials and Methods

### Ethic statements

All fish are housed in the fish facility of our laboratory, which was built according to the local animal welfare standards. All animal procedures were performed in accordance with French and European Union animal welfare guidelines.

### Gene identification

Calcium binding protein members in *Danio rerio* were identified based on orthology predictions in Ensembl using the Zv9 (GCA_000002035.2) assembly. To confirm gene identities we performed multispecies alignments within the 5 teleost species *O. latipes*, *T. rubipres*, *G. aculeatus*, *T. nigroviridis* and *D. rerio* and used the following genes: *cabp1a* (ENSDARG00000033411), *cabp1b* (ENSDARG00000019990), *cabp2a* (ENSDARG00000052016), *cabp2b* (ENSDARG00000052277), *cabp4a* (ENSDARG00000008866), *cabp5a* (ENSDARG00000002576), *cabp5b* (ENSDARG00000028485), *cabp7a* (ENSDARG00000060846), *cabp7b* (ENSDARG00000078272), *caln1* (*cabp8a*) (ENSDARG00000088898), *caln2* (*cabp8b*) (ENSDARG00000069766).

In the case of *cabp4* there was just one ortholog annotated in the zebrafish Zv9 assembly. As there were two paralogs present in all other teleost fish species we used the cDNA sequence of *cabp4* (2of2) (ENSTNIG00000005250) of Tetraodon and performed a BlastN search against the EST selection of NCBI. As a result we found a cDNA clone (BG307419, fm03g10.y1, IMAGE:4145466) that showed 77% sequence similarity to the Tetraodon sequence and named it *cabp4b*.

### Phylogenetic analysis

The protein sequences of *Danio rerio* and *Homo sapiens* CaBPs were obtained from Ensembl. For *cabp7b*, there was no annotated start codon and we were not able to identify a suitable translational start in the genomic upstream sequence. For *cabp4a* neither start nor stop codon were annotated. By including the flanking genomic region in the search for an ORF, both could potentially be identified upstream and downstream of the annotated gene. For the newly identified *cabp4b* the whole sequence given as BG307419 was used and translated in frame +1. Alignment of the sequences was produced using MUSCLE online at the European Bioinformatics Insitute (EBI) [23]. The result of the algorithm was visualized using Jalview [24]. A phylogenetic tree was assembled using BioNJ online at phylogeny.fr performing 1000 bootstraps and using the Jones-Taylor-Thornton matrix. This distance-based algorithm is claimed to be well suited for comparison of sequences with high substitution rates [25], [26]. The tree was visualized using the open source software Dendroscope [27]. To identify the functional domains within the *cabp4b* ORF we used the ExPASy Prosite database from the Swiss Institute of Bioinformatics (SIB, www.expasy.org).

### Fish husbandry and strains used

Breeding and raising of zebrafish followed standard protocols [28]. Zebrafish embryos were treated with 0.0045% 1-Phenyl-2-Thiourea (PTU) in medium after gastrulation to prevent pigment formation. For one color whole mount *in situ* hybridisation Tupfel long fin fish were used. The transgenic Tg(Vsx2:GFP) [18] and Tg(Ptf1A:GFP) [17] lines were a kind gift of Lucia Poggi and Jochen Wittbrodt; the Tg(Brn3C:memGFP) [19] line was obtained from Herwig Baier.

### Molecular cloning

For generating an anti-sense probe for *cabp1a*, we ordered the clone IRBOp991H0750D from Imagenes. Subsequently, the coding fragment was cloned into the pBluescript KS vector (Invitrogen). Following linearization of the plasmid with HindIII and DNA clean-up, a Digoxigenin (DIG)-labeled antisense probe was generated using T7 RNA polymerase. Following DNaseI treatment, the synthesized probe was purified using the NucleoSpin® RNA II Kit (Macherey-Nagel).

The same procedure was applied for the synthesis of the anti-sense probes of *cabp2a* (IMAGp998N1115278Q(2)); into pAMP1, linearization with KpnI, transcription with SP6 polymerase; *cabp2b* (IMAGp998G1716318Q); into pBluescript Sk-, linearization with EcoRV, transcription with T7 polymerase; *cabp4a* (IMAGp998D0510667Q(4)); into pBluescript Sk-, linearization with BamHI/NotI, transcription with T7 polymerase; *cabp5a* (IRBOp991H021D); into pBluescript Sk-, linearization with NcoI, transcription with T7 polymerase; *cabp5b* (IMAGp998E2112463Q(5+3)b) into pAMP1, linearization with EcoRI, transcription with SP6 polymerase. For *cabp1b*, *cabp4b*, *cabp7a*, *cabp8a* and *cabp8b*, cDNA fragments were PCR amplified from zebrafish total cDNA using the following primers (5′prime to 3′prime): CaBP1b_fwd: CTATGGGAAACTGTGTTAAATCGCCGC; CaBP1b-rev: TTATCTCTAGCGAGACATCATTCGCAC; CaBP4b_fwd: CATCCACGATACACCATGGCAC; CaBP4b_rev: TCCACAGAGATCTTCCCATCGC; CaBP7a_fwd: AAGTGGAGGAGATCCGTGAAGC; CaBP7a_rev: ATCAAGCTCTTTCGCACACAGG; CaBP8a_fwd: AAGGAGATGAGGGGCTAGGGAG; CaBP8a_rev: CACACACGTCTGACGGTTCTTC; CaBP8b_fwd: GGAGCGAGCTTTTCACCTGATG; CaBP8b_rev: TGGTGAGATGGTCTCTGAAGGC. PCR fragments were cloned into the pCRII-TOPO vector (Invitrogen) according to manufacturers' instructions. All plasmids used were confirmed by sequencing.

### In situ hybridisation

*In vitro* transcription of Digoxigenin-labeled probes was performed using the RNA Labeling Kit (Roche Diagnostics Corporation) according to manufacturer's instructions. Dechorionated embryos at the appropriate developmental stage(s) were fixed in 4% paraformaldehyde in 1× phosphate buffered saline (pH 7.4) for 2 h at room temperature and whole-mount *in situ* hybridisation was performed as previously described [29].

### Antibody staining

After *in situ* hybridisation staining, whole mount zebrafish embryos from Tg(Vsx2:GFP), Tg(Brn3C:memGFP) and Tg(Ptf1a:GFP) transgenic lines were washed twice in 1× PBS/0.1% Tween-20 solution. Subsequently, they were incubated 1 h at room temperature in 10% Normal Goat Serum (Invitrogen), 1% DMSO in PBS-Tw blocking solution followed by overnight incubation with 1/1000 dilution of chicken primary anti-GFP antibody (Genetex). The Alexa Fluor 488 secondary antibody goat anti-chicken IgG (1/500, Molecular probes) and a 1/500 dilution of DAPI (50 µg/µL) in blocking solution were added overnight. After 5× washing in wash buffer (1× PBS/0.1% Tween-20) microscopy analysis was performed.

### Vibratome sections and confocal microscopy

After staining, whole-mount embryos were washed twice in 0,1% Tween in PBS 1×. Afterwards, the samples were embedded in gelatin/albumin with 4% of Glutaraldehyde and sectioned (20 µm) on a VT1000 S vibrating blade microtome (Leica). The sections were analyzed on a Leica Upright Widefield epifluorescence microscope and a Zeiss LSM710 confocal microscope (Zeiss). Images were processed using ImageJ, Adobe Photoshop and Adobe Illustrator software.

## References

[1] Berridge MJ (1998) Neuronal calcium signaling. Neuron 21: 13–26. 9697848 10.1016/s0896-6273(00)80510-3

[2] Catterall WA, Few AP (2008) Calcium channel regulation and presynaptic plasticity. Neuron 59: 882–901. 18817729 10.1016/j.neuron.2008.09.005

[3] Mochida S, Few AP, Scheuer T, Catterall WA (2008) Regulation of presynaptic Ca(V)2.1 channels by Ca2+ sensor proteins mediates short-term synaptic plasticity. Neuron 57: 210–216. 18215619 10.1016/j.neuron.2007.11.036

[4] Greer PL, Greenberg ME (2008) From synapse to nucleus: calcium-dependent gene transcription in the control of synapse development and function. Neuron 59: 846–860. 18817726 10.1016/j.neuron.2008.09.002

[5] McCue HV, Haynes LP, Burgoyne RD (2010) The diversity of calcium sensor proteins in the regulation of neuronal function. Cold Spring Harb Perspect Biol 2: a004085. 20668007 10.1101/cshperspect.a004085 PMC2908765

[6] Dolmetsch RE, Pajvani U, Fife K, Spotts JM, Greenberg ME (2001) Signaling to the nucleus by an L-type calcium channel-calmodulin complex through the MAP kinase pathway. Science 294: 333–339. 11598293 10.1126/science.1063395

[7] Kawasaki H, Nakayama S, Kretsinger RH (1998) Classification and evolution of EF-hand proteins. Biometals 11: 277–295. 10191494 10.1023/a:1009282307967

[8] McCue HV, Haynes LP, Burgoyne RD (2010) Bioinformatic analysis of CaBP/calneuron proteins reveals a family of highly conserved vertebrate Ca2+-binding proteins. BMC Res Notes 3: 118. 20426809 10.1186/1756-0500-3-118 PMC2873350

[9] Haeseleer F, Sokal I, Verlinde CL, Erdjument-Bromage H, Tempst P, et al (2000) Five members of a novel Ca(2+)-binding protein (CABP) subfamily with similarity to calmodulin. J Biol Chem 275: 1247–1260. 10625670 10.1074/jbc.275.2.1247 PMC1364469

[10] Wu YQ, Lin X, Liu CM, Jamrich M, Shaffer LG (2001) Identification of a human brain-specific gene, calneuron 1, a new member of the calmodulin superfamily. Mol Genet Metab 72: 343–350. 11286509 10.1006/mgme.2001.3160

[11] Mikhaylova M, Sharma Y, Reissner C, Nagel F, Aravind P, et al (2006) Neuronal Ca2+ signaling via caldendrin and calneurons. Biochim Biophys Acta 1763: 1229–1237. 17055077 10.1016/j.bbamcr.2006.08.047

[12] Haeseleer F, Imanishi Y, Maeda T, Possin DE, Maeda A, et al (2004) Essential role of Ca2+-binding protein 4, a Cav1.4 channel regulator, in photoreceptor synaptic function. Nat Neurosci 7: 1079–1087. 15452577 10.1038/nn1320 PMC1352161

[13] Rieke F, Lee A, Haeseleer F (2008) Characterization of Ca2+-binding protein 5 knockout mouse retina. Invest Ophthalmol Vis Sci 49: 5126–5135. 18586882 10.1167/iovs.08-2236 PMC2804972

[14] Haynes LP, McCue HV, Burgoyne RD (2012) Evolution and functional diversity of the Calcium Binding Proteins (CaBPs). Front Mol Neurosci 5: 9. 22375103 10.3389/fnmol.2012.00009 PMC3284769

[15] Wittbrodt JMA, Scharti M (1998) More genes in fish? BioEssays 20: 511–515.

[16] Sprague J, Bayraktaroglu L, Clements D, Conlin T, Fashena D, et al (2006) The Zebrafish Information Network: the zebrafish model organism database. Nucleic Acids Res 34: D581–585. 16381936 10.1093/nar/gkj086 PMC1347449

[17] Jusuf PR, Harris WA (2009) Ptf1a is expressed transiently in all types of amacrine cells in the embryonic zebrafish retina. Neural Dev 4: 34. 19732413 10.1186/1749-8104-4-34 PMC2746205

[18] Vitorino M, Jusuf PR, Maurus D, Kimura Y, Higashijima S, et al (2009) Vsx2 in the zebrafish retina: restricted lineages through derepression. Neural Dev 4: 14. 19344499 10.1186/1749-8104-4-14 PMC2683830

[19] Xiao T, Roeser T, Staub W, Baier H (2005) A GFP-based genetic screen reveals mutations that disrupt the architecture of the zebrafish retinotectal projection. Development 132: 2955–2967. 15930106 10.1242/dev.01861

[20] Zeitz C, Kloeckener-Gruissem B, Forster U, Kohl S, Magyar I, et al (2006) Mutations in CABP4, the gene encoding the Ca2+-binding protein 4, cause autosomal recessive night blindness. Am J Hum Genet 79: 657–667. 16960802 10.1086/508067 PMC1592568

[21] Vihtelic TS, Fadool JM, Gao J, Thornton KA, Hyde DR, et al (2005) Expressed sequence tag analysis of zebrafish eye tissues for NEIBank. Mol Vis 11: 1083–1100. 16379021

[22] Force A, Lynch M, Pickett FB, Amores A, Yan YL, et al (1999) Preservation of duplicate genes by complementary, degenerative mutations. Genetics 151: 1531–1545. 10101175 10.1093/genetics/151.4.1531 PMC1460548

[23] Edgar RC (2004) MUSCLE: multiple sequence alignment with high accuracy and high throughput. Nucleic Acids Res 32: 1792–1797. 15034147 10.1093/nar/gkh340 PMC390337

[24] Waterhouse AM, Procter JB, Martin DM, Clamp M, Barton GJ (2009) Jalview Version 2–a multiple sequence alignment editor and analysis workbench. Bioinformatics 25: 1189–1191. 19151095 10.1093/bioinformatics/btp033 PMC2672624

[25] Dereeper A, Guignon V, Blanc G, Audic S, Buffet S, et al (2008) Phylogeny.fr: robust phylogenetic analysis for the non-specialist. Nucleic Acids Res 36: W465–469. 18424797 10.1093/nar/gkn180 PMC2447785

[26] Gascuel O (1997) BIONJ: an improved version of the NJ algorithm based on a simple model of sequence data. Mol Biol Evol 14: 685–695. 9254330 10.1093/oxfordjournals.molbev.a025808

[27] Huson DH, Richter DC, Rausch C, Dezulian T, Franz M, et al (2007) Dendroscope: An interactive viewer for large phylogenetic trees. BMC Bioinformatics 8: 460. 18034891 10.1186/1471-2105-8-460 PMC2216043

[28] Westerfield M The zebrafish book. A guide for the laboratory use of zebrafish (Danio rerio). Univ of Oregon Press, Eugene 4th ed.

[29] Thisse C, Thisse B (2008) High-resolution in situ hybridisation to whole-mount zebrafish embryos. Nat Protoc 3: 59–69. 18193022 10.1038/nprot.2007.514

